# 

**Published:** 1822-09-01

**Authors:** 


### Contents

OF

### No. 10, Vol. III, for SEPTEMBER, 1822.

Art. I.

An Inquiry into the Opinions, ancient and modern, concerning Life and Organization. By John Barclay, M.D. 225

II.

Lectures on the Structure and Physiology of the Male urinary Organs. By James Wilson, F.R.S. - - - - - 260

III.

De l'Alienation Mentale des Nouvelles Accouchées, et des Nourrices. By M. Esquirol, M.D. - - - - - - - 269

IV.

A Treatise on Diseases of the Nervous System. By A. C. Prichard, M. D. (Concluded) - - - - - - - - - 277

V.

Essays on Surgery and Midwifery; with Practical Observations. By James Barlow. (Concluded) - - - - - 306

VI.

Further Observations on Strictures of the Rectum; with M. Boyer's paper on Spasmodic Stricture of the Sphincter Ani - - - - - - - - - - - - - - - - - 326

VII.

A Treatise on the Nature and Treatment of Scrofula. By Eusebius Lloyd. (Concluded from page 174). - - 333

VIII.

A System of Surgical Anatomy. Part 1. On the Structure of of the Groin, Pelvis, and Perineum, &c. By William Anderson Esq. of New York. - - - - - - - - 357

IX.

Dr. Chisholm on various Diseases - - - - - - - - - 376

X.

Medico-Chirurgical Transactions, Vol. xii - - - - - - 386

XI.

Quarterly Periscope, or Spirit of the Public Journals 393

Art. 1. Drs. Armstrong and Autenrieth on Fever- - - - 393
2. Reunion of Separated Bone - - - - - - - - 400
3. Metastases, curious Cases of - - - - - - - - - 401
4. Mr. Todd's Operation for Axillary Aneurism - - 402
5. M. Dubois on Malignant Ulcer of the Penis - - 405
6. Dr. Gibney on Oil of Turpentine - - - - - - 406
7. Dr. Zolickoffer on Rheumatism - - - - - - - 406

8. Congenital Dyspepsia - - - - - - - - - - - 407
9. Mr. Lizars on Hernia - - - - - - - - - - - 408
10. M. Dubois's Case of Hernia - - - - - - - - 410
11. Dr. Hosack on Emetics in Constipation - - - - 410
12. Mr. Thompson on Belladonna - - - - - - - 412
13. Dr. Kennedy's Case of Poisoning - - - - - - 413
14. Venereal Affection of the Heart - - - - - - - 413
15. Dr. Page or Tobacco in the Phlegmasiæ - - - 414
16. Case of dislocated Femur - - - - - - - - - 415
17. Introduction of Foreign Matters into the Blood - 416
18. Carcinoma mistaken for Aneurism - - - - - - 420
19. Malaria, in St. James's Park - - - - - - - - 422
20. Traumatic Tetanus - - - - - - - - - - - - 423
21. Over-dose of Digitalis - - - - - - - - - - 425
22. Dr. M'Carthy on Ascites - - - - - - - - - 425
23. Mr. Plumbe's Case of Uterine Contraction - - - 426
24. M. Richerand on Cancer of the Lip - - - - - 427
25. Mr. Wray and Dr. Copland on Cold Affusion - - 428
26. Remarkable Wound of the Brain - - - - - - 428
27. Counter-irritation - - - - - - - - - - - - 429
28. Hydrophobia - - - - - - - - - - - - - - 429
29. Mr. Bryant's Case of Obliterated Iliac - - - - 431
30. Dr. Hosack on Phlegmatia Dolens - - - - - - 432
31. Mr. Sprague on Poisoning by Opium - - - - - 433
32. Mr. Harrison's Case of double Hare-lip - - - - 433
33. Dr. Hebreart on the Pathology of the Brain - - 434

XII.

Bibliographical Record - - - - - - - - - - - - - - 441

XIII.

EXTRA LIMITES.

Art. 1. Mr. Money on Oleum Terebinthinæ - - - - - - 446
2. Report of the Surgeon-Apothecaries - - - - - 453
3. Dr. Johnson's Case of Visceral Disease - - - - 456
4. Mr. Stevenson on Amaurosis - - - - - - - - 458

XIV.

CORRESPONDENCE, INTELLIGENCE, &c.

Transatlantic Correspondence - - - - - - - - - - 460

Medical Intelligence - - - - - - - - - - - - - - 460

Apothecaries' Act - - - - - - - - - - - - - - - 461

Dr. Pemberton's Death - - - - - - - - - - - - - 461

Dr. Parry's Elements of Pathology - - - - - - - - - 461

XV.

List of Additional Subscribers - - - - - - - - - - 463

---

P.S. Several Subscribers Names, which arrived after the 15th August, and when the List of "Additional Subscribers" had been composed, are unavoidably held over till our next.

